# Supplementary material for: The Construction and Exploration of a Comprehensive MicroRNA Centered Regulatory Network in Foxtail Millet (Setaria italica L.)
Source: Front Plant Sci. 2022 May 6;13:848474. doi: 10.3389/fpls.2022.848474 (PMC9121102; doi:10.3389/fpls.2022.848474)
Supplement: Supplementary file 4 [file Data_Sheet_4.zip › Sit-miR398a_Seita.1G154000.1_99_TPlot.pdf]

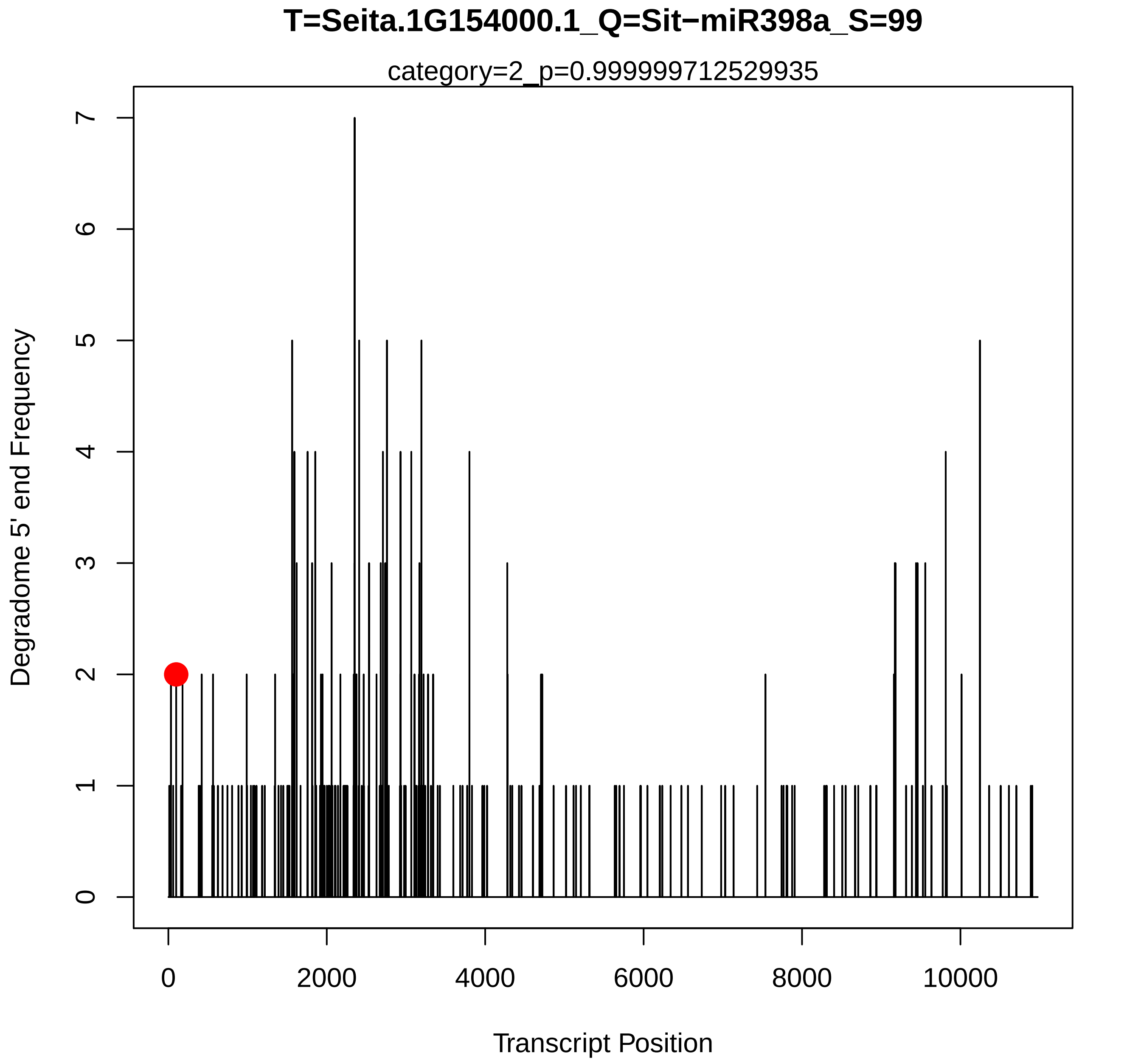

T=Seita.1G154000.1_Q=Sit−miR398a_S=99
category=2_p=0.999999712529935
Degradome 5' end Frequency
0
1
2
3
4
5
6
7
0
2000
4000
6000
8000
10000
Transcript Position
